# Supplementary material for: Extremely high magnetoresistance and conductivity in the type-II Weyl semimetals WP2 and MoP2
Source: Nat Commun. 2017 Nov 21;8:1642. doi: 10.1038/s41467-017-01758-z (PMC5696372; doi:10.1038/s41467-017-01758-z)
Supplement: Supplementary file 1 — Supplementary Information [file 41467_2017_1758_MOESM1_ESM.pdf]

Supplementary Note 1

Type-II Weyl semimetal in WP_2. The energy dispersions along high symmetry lines show the semimetallic character of WP_2 as shown in Supplementary Fig. 1a. The hole and electron pockets mainly locate around *X* and *Y* point, respectively in the in the Brillouin zone (BZ), which is consistent with the previous report.[1] Same as the GGA calculation in Ref. 1, we also find the two classes of type-II Weyl points in the k_z=0 plane in MBJ approximation, which lye 0.3 and 0.5 eV below the Fermi level (labeled as W1 and W2 in Supplementary Fig. 1b). The coordinates of the two Weyl points are (0.2560/Å, 0.2939/Å, 0) and (0.2606Å, 0.3347/Å, 0) in Cartesian coordinates. Via time reversal and mirror reflections we can get the other equivalent Weyl points. The Weyl points are further confirmed by the monopole of the Berry curvature around the gapless points. Both W1 and W2 have positive chirality in the positive zone of the BZ, and the mirror reflection changes their signs, see Supplementary Fig. 1c.

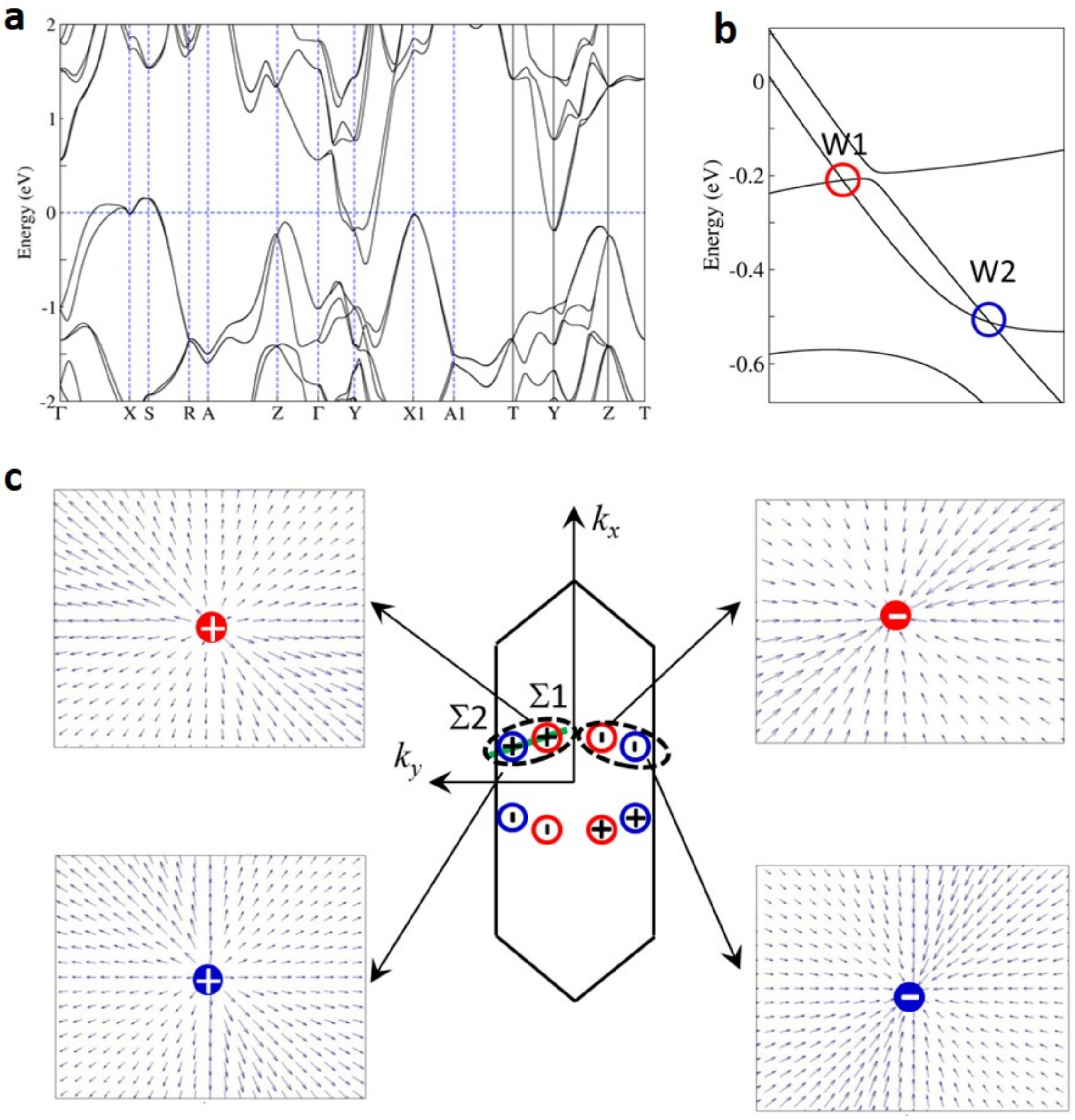

Supplementary Figure 1: Band structure and Weyl points in WP_2 from MBJ calculation. a Energy dispersions along high symmetry lines. **b** Energy dispersion crossing two Weyl points in the k_z = 0 plane. **c** Location of the Weyl points and Berry curvature distribution around the Weyl points in k_z = 0 plane. The green line is the *k*-path used in **b**.

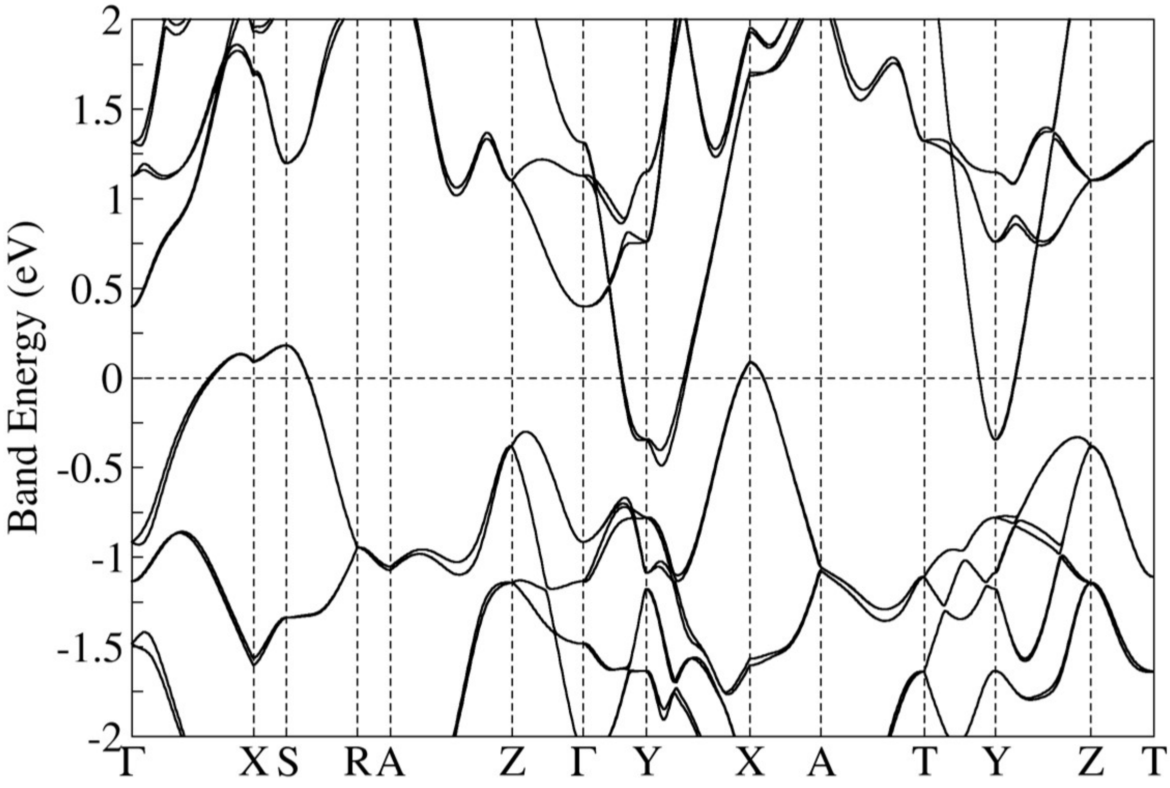

Supplementary Figure 2: **Band structure of MoP_2 from MBJ calculation showing energy dispersions along high symmetry lines.**

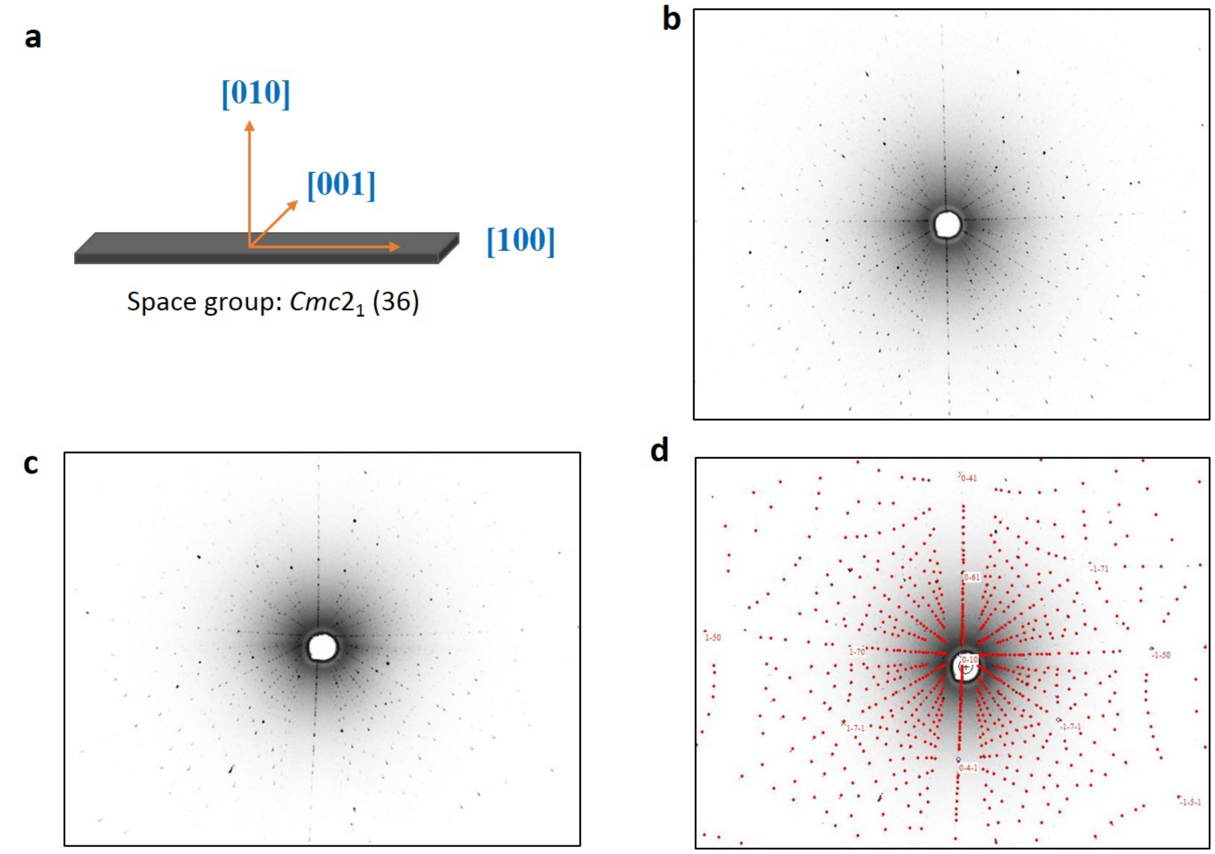

Supplementary Figure 3: White beam backscattering Laue X-ray diffraction method at room temperature of C3. a Schematic of the crystal orientation of WP_2. **b** A diffraction pattern on shining the X-ray beam along *b*-axis. The well-defined sharp Laue spots indicate excellent quality of the grown crystals without any twining or domain. We rotate the crystal ~ 5° (in order to obtain high symmetry points in the diffraction pattern) perpendicular to the X-ray beam and the new pattern is presented in **c**. Data fits with orthorhombic space group $Cmc2_1$ (36) with lattice parameters $a = 3.1677$ Å, $b = 11.1652$ Å and $c = 4.9751$ Å. **d** Superimposed theoretically simulated pattern (red dots). We find that the rectangular crystals naturally grow with length along *a*-axis and the flat surface is normal to *b*-axis.

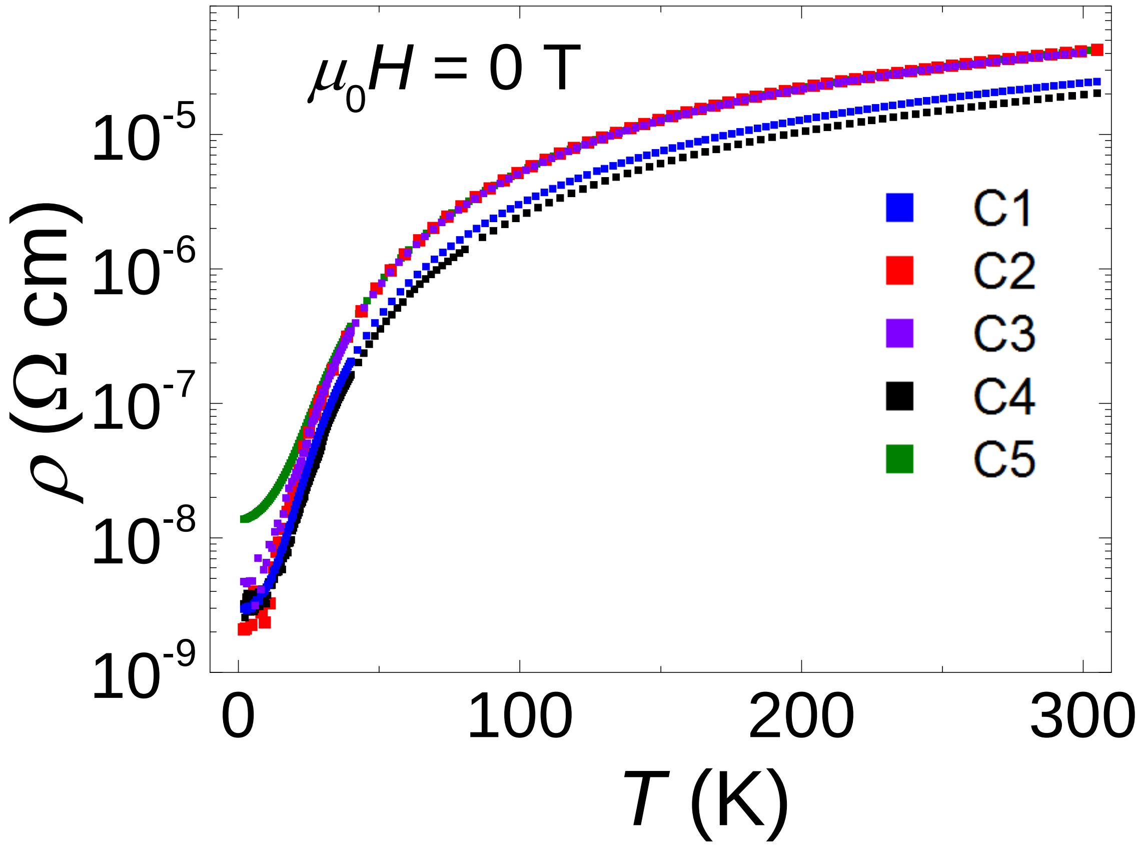

Supplementary Figure 4: Zero-field longitudinal resistivity as a function of temperature. The current is applied *a*-axis. The resistivity is plotted in the log-scale. All the measured crystals show very low resistivity ~ 3-4 nΩcm at 2 K with the exception of crystal C5 where the 2 K resistivity is 12 nΩcm.

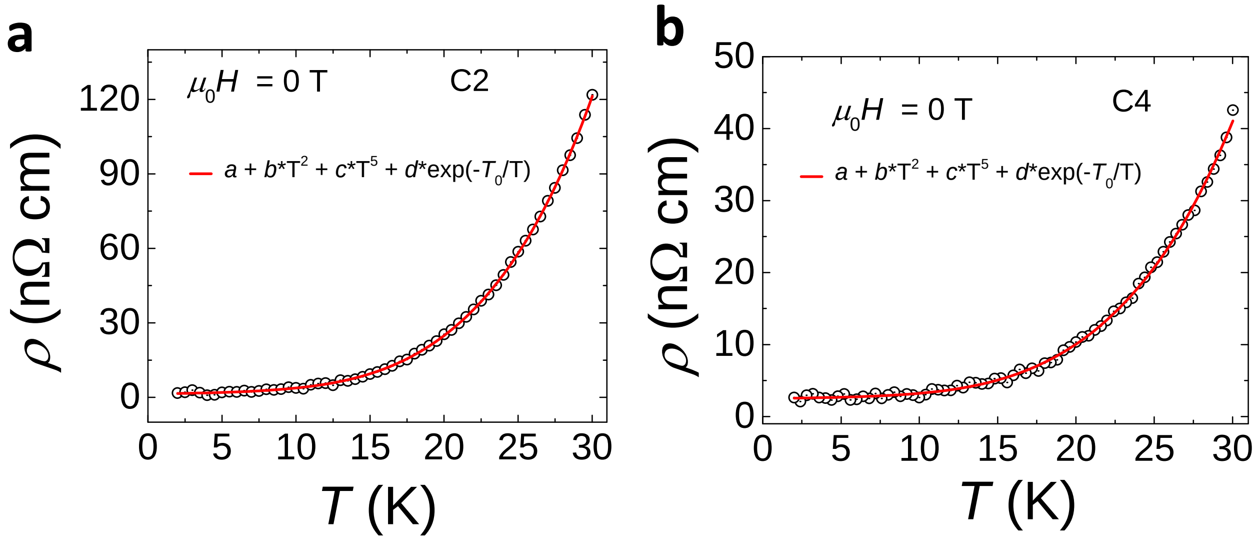

Supplementary Figure 5: Low temperature resistivity fitting of WP_2. **a** and **b** shows the fitting of low temperature resistivity data at 0 T considering the electron-defect (temperature independent), electron-electron (T^2), electron-phonon (T^5) and phonon drag (exponential) contributions.

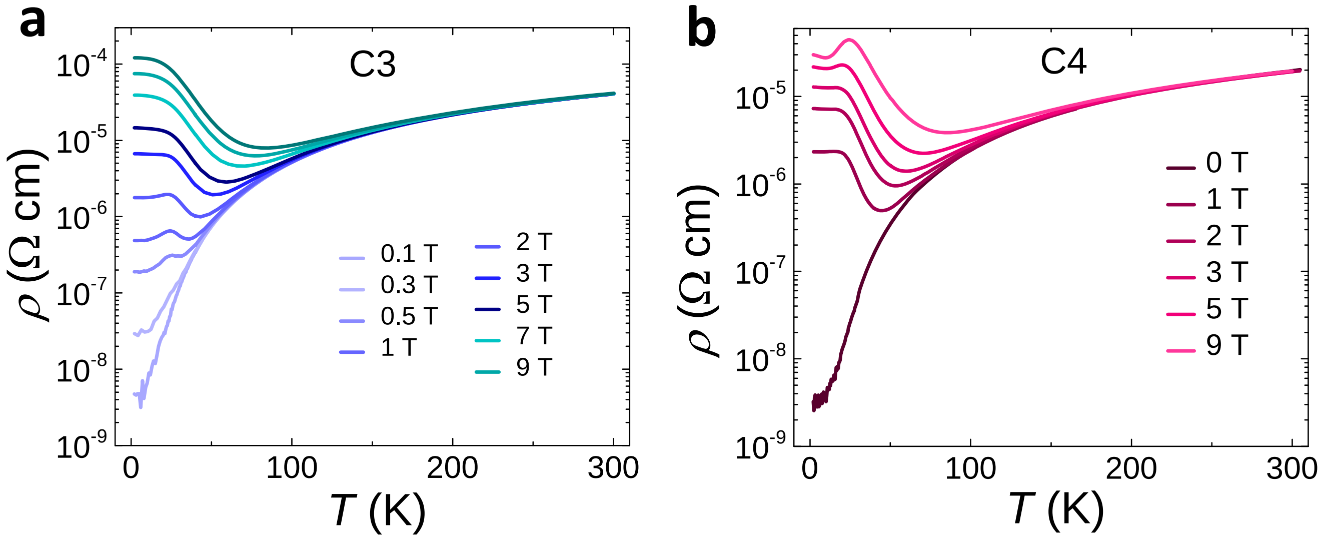

Supplementary Figure 6: $\rho(T)$ data of WP_2 as a function of magnetic field. **a** and **b** shows the resistivity of crystals C3 and C4, respectively at increasing magnetic field up to 9 T exhibiting extremely large MR

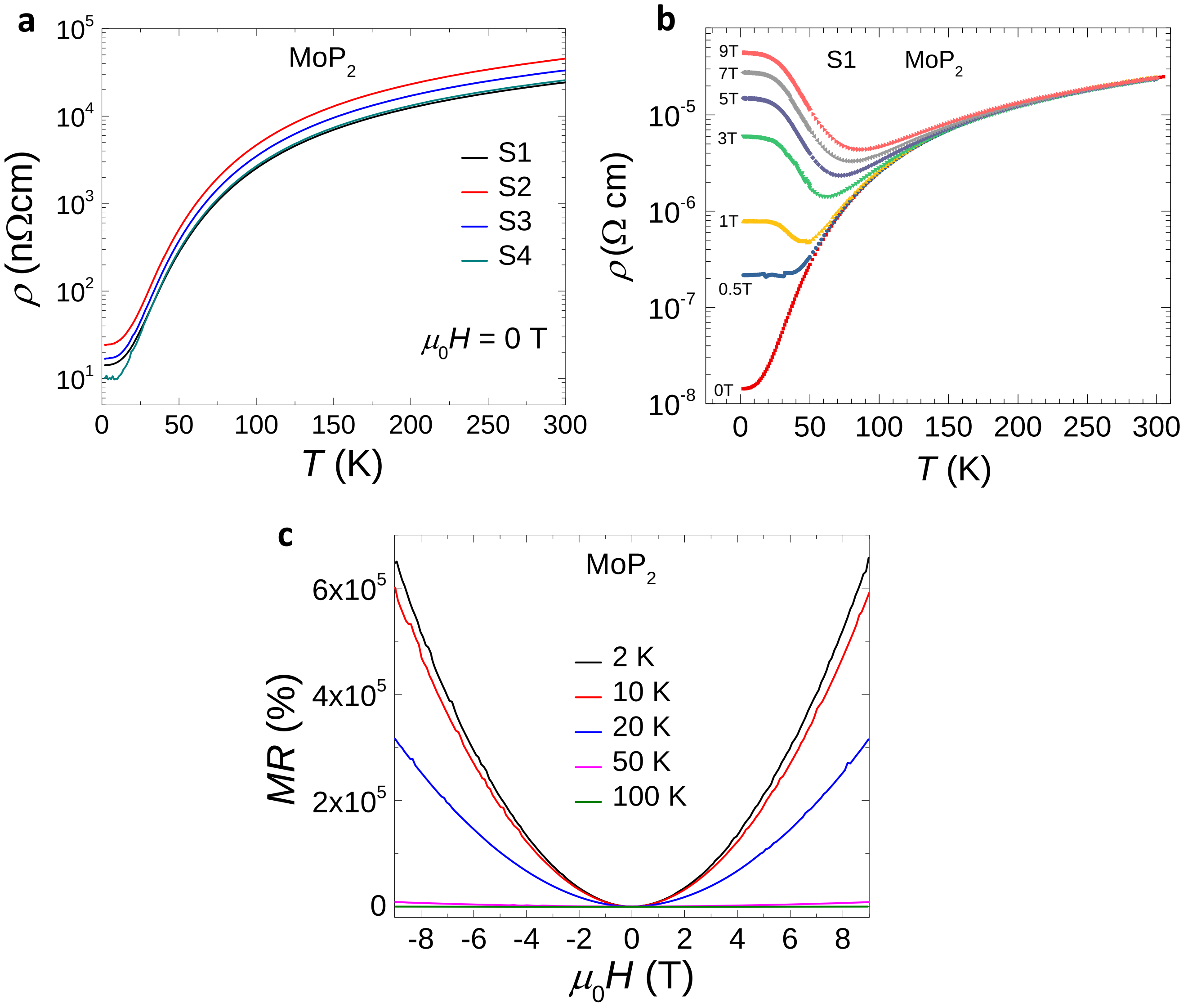

Supplementary Figure 7: ρ(T) and $\rho(\mu_0 H)$ data of MoP_2. a Zero field resistivity of four different crystals of MoP_2 with ρ_0 between 10-24 nΩ cm. **b** $\rho(T)$ data for crystal S1 at magnetic fields up to 9 T. **c**. $\rho(\mu_0 H)$ data of crystal S4 showing an MR value of 6.5×10^5 % at 2 K and 9 T.

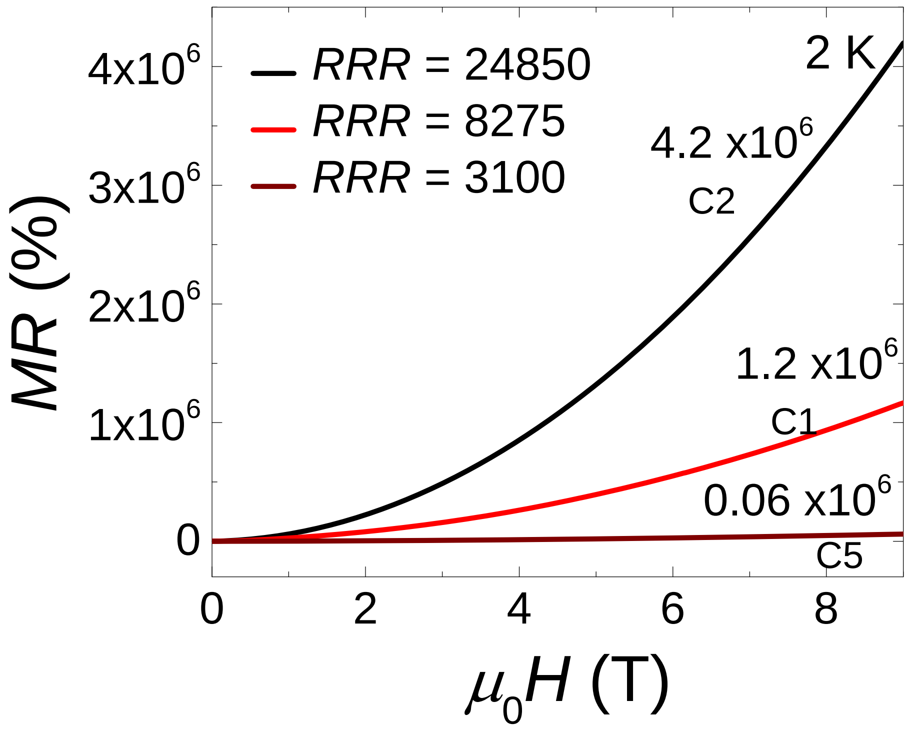

Supplementary Figure 8: *MR* of three WP_2 crystals at 2 K with different *RRR* values. Crystal with the highest *RRR* exhibits the largest *MR*.

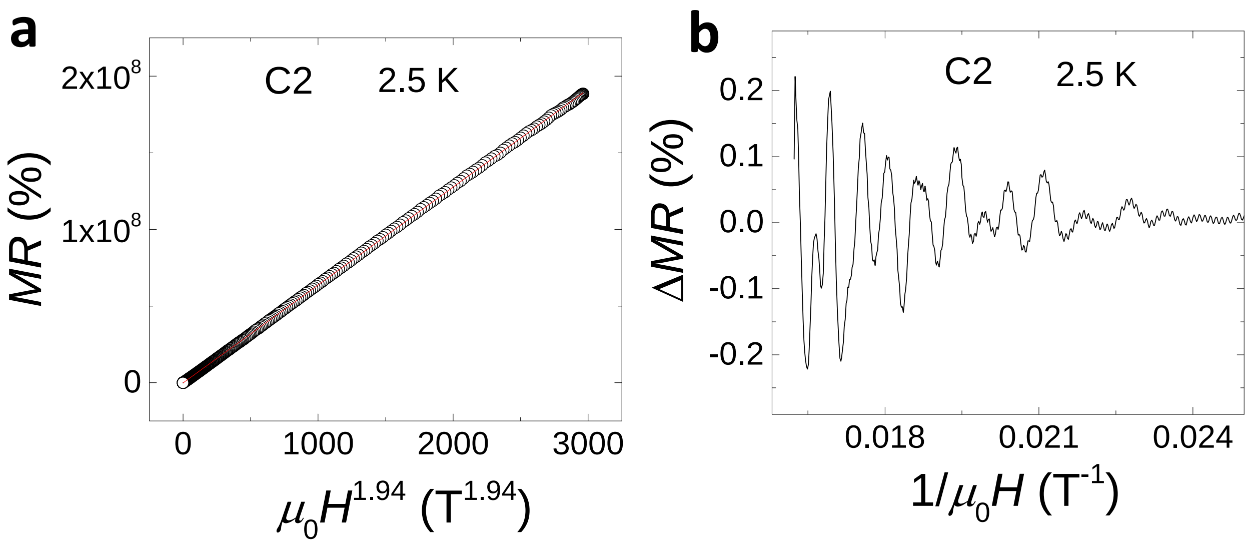

Supplementary Figure 9: *MR* of WP_2 in 63 T field. a *MR* as a function of $B^{1.94}$ along with the linear fit (red line)**. b** Oscillation amplitude showing percentage change in *MR* at 2.5 K.

Supplementary Note 2

WP_2 as a megagauss sensor. The *MR* data is described by a near quadratic field dependence, $MR \propto B^{1.94}$, up to the maximum field as shown in Fig. 3d. This makes WP_2 an ideally suited material for accurate magnetic field sensors which can be used in the megagauss regime. The limit of the error in the measured magnetic field by this method would be set by amplitude of the quantum oscillations at very high magnetic field. We observe that the amplitude of the quantum oscillations is very small at 63 T and hence it introduces a very small error (0.2 %. See Fig. S9) in a *B* measurement. One of the most efficient megagauss sensor candidates proposed earlier was based on the linear *MR* in silver chalcogenide. However, the *B*-dependent *MR* is not perfectly linear particularly at very high fields.[2] It is important to note that compensated semimetals like WTe_2 [3], LaBi [4] *etc.* also exhibit a quadratic *MR* with *B* but because of the large amplitude of quantum oscillations cannot be used as megagauss sensors.

WP_2

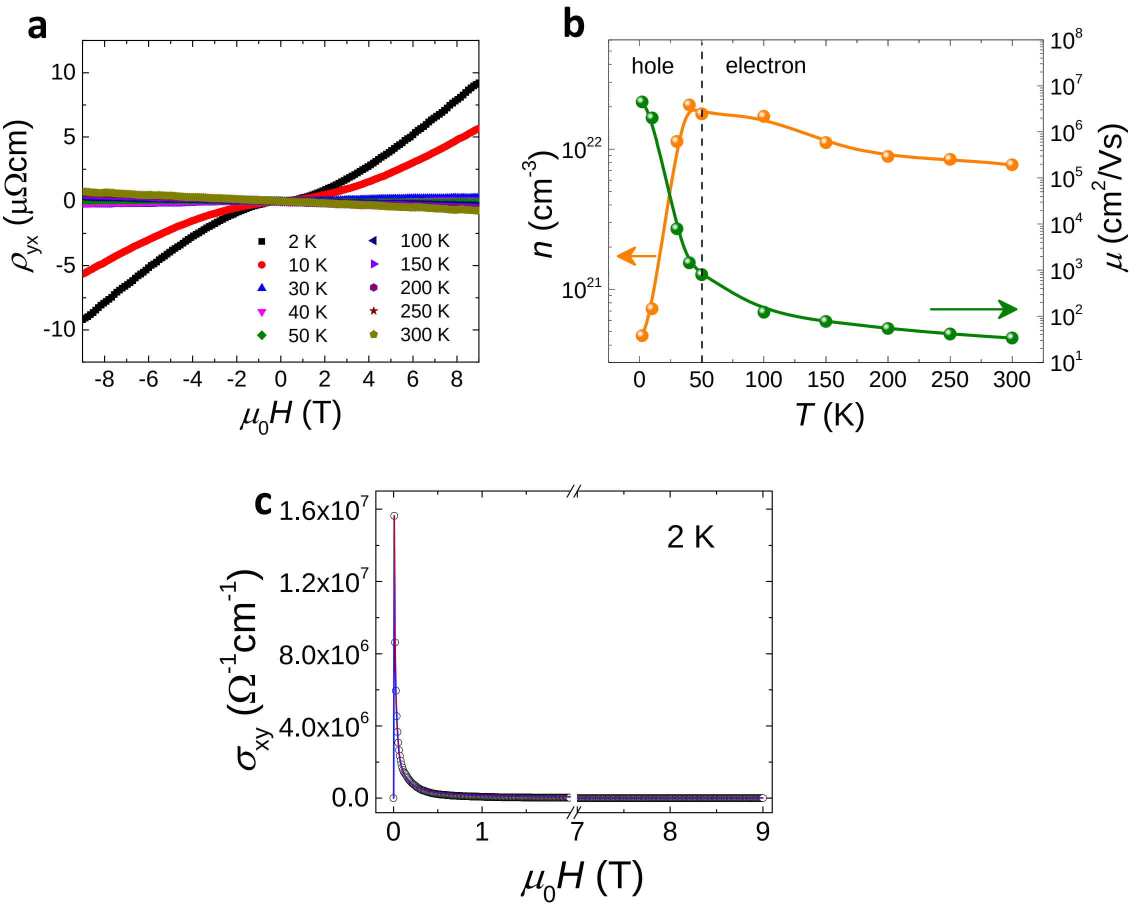

Supplementary Figure 10: Hall resistivity and calculated carrier density and mobility of WP_2. a shows the Hall resistivity of WP_2 at different temperatures. **b** shows the average carrier concentration and mobility obtained from single band model. At high temperature electrons are the dominant charge carriers which changes to holes below 50 K. **c** The Hall conductivity at 2 K fitted to the equation: $\sigma_{xy} = \left[n_h \mu_h^2 \frac{1}{1+(\mu_h B)^2} - n_e \mu_e^2 \frac{1}{1+(\mu_e B)^2}\right] eB$. The values of n_h (hole concentration) and n_e (electron concentration) obtained from the fitting are 1.5×10^{20} cm^{-3} and 1.4×10^{20} cm^{-3}, respectively. The hole and electron mobility (μ_h and μ_e) is 1.65×10^6 cm^2/Vs. The Hall conductivity was calculated from the equation: $\sigma_{xy} = \frac{\rho_{yx}}{\rho_{xy}^2 + \rho_{xx}^2}$.

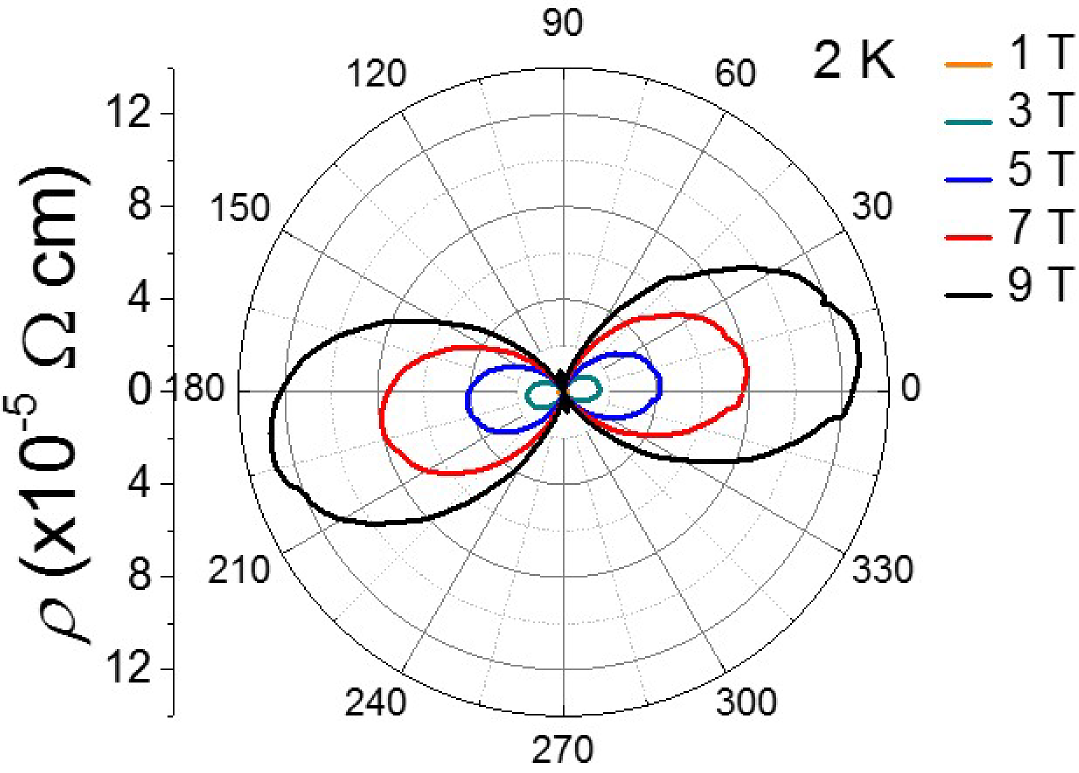

Supplementary Figure 11: Anisotropic resistivity of WP_2 (C3). Resistivity as a function of θ at various magnetic fields. The current was applied along a-axis and the magnetic field was rotated in the *bc*-plane. The *b*-axis is 10° away from the 0° due to sample misalignment. The accurate alignment of the crystal is difficult due to its needle shape. The anisotropy in the *MR* for the field directions along b-axis and c-axis is 2.5 order of magnitude. Surprisingly, the anisotropy is much higher than in WTe_2 which despite having a 2D van der Waals structure show only one order of magnitude anisotropy in *MR*.[5] WTe_2 is therefore 3D electronically. In contrast, in WP_2 the hole-pockets are open (2D) and run along the *b*-axis which causes an extremely large anisotropic *MR*.[6]

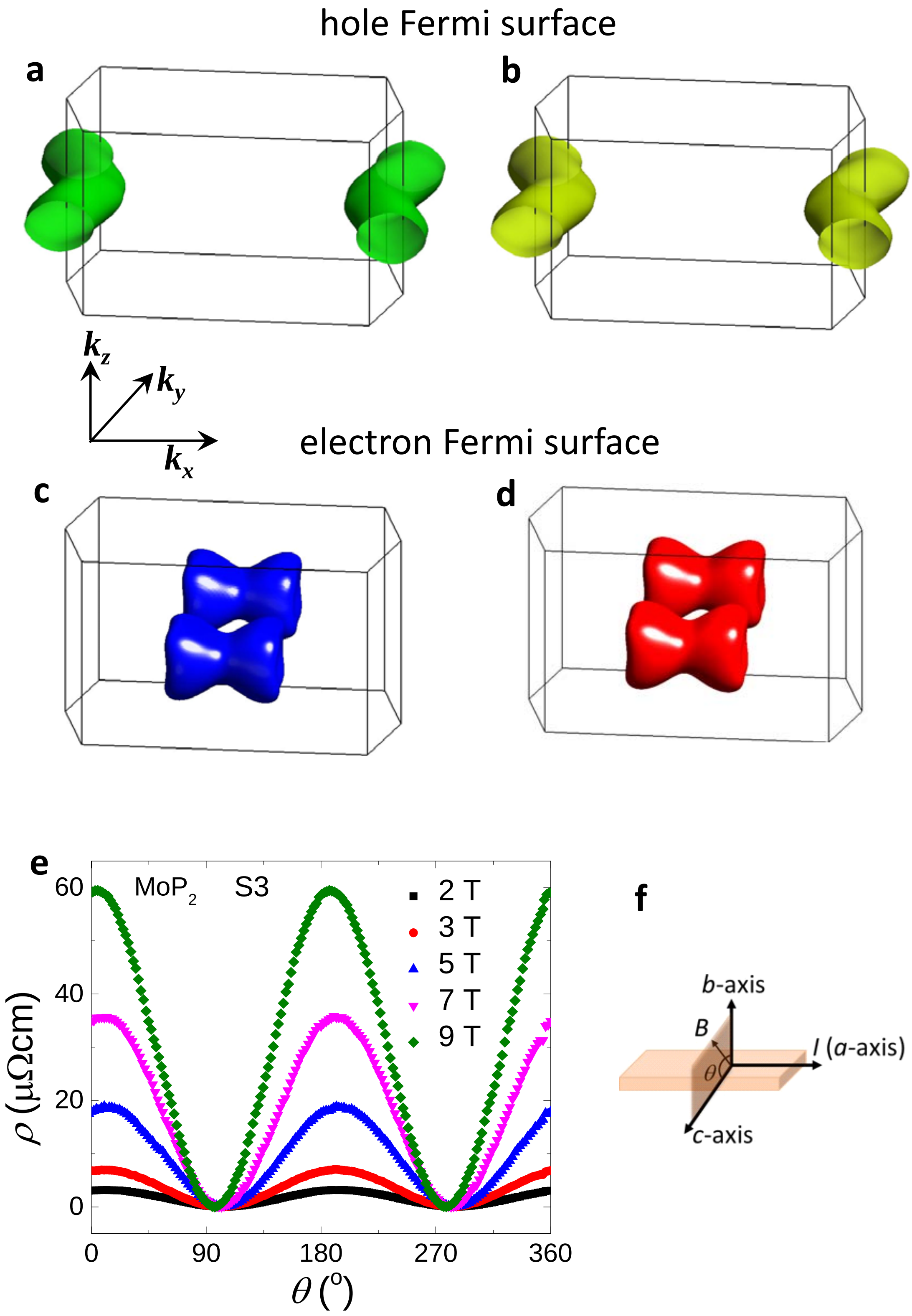

Supplementary Figure 12: Fermi surface topology and anisotropic *MR* in MoP_2. Spaghetti-like open hole Fermi surfaces located around *X*-point in the BZ, extending along the *c*-axis in **a** and **b**. Bow-tie-like closed electron Fermi surfaces located around Y-point in the BZ in **c** and **d**. **e** shows the anisotropy in the resistivity due to the Fermi surface topology. *MR* is the maximum and minimum when *B* is parallel to the *b*- and *c*-axis, respectively. *I* is applied along the *a*-axis. **f** The geometry of the device with marked axes, magnetic field and current directions.

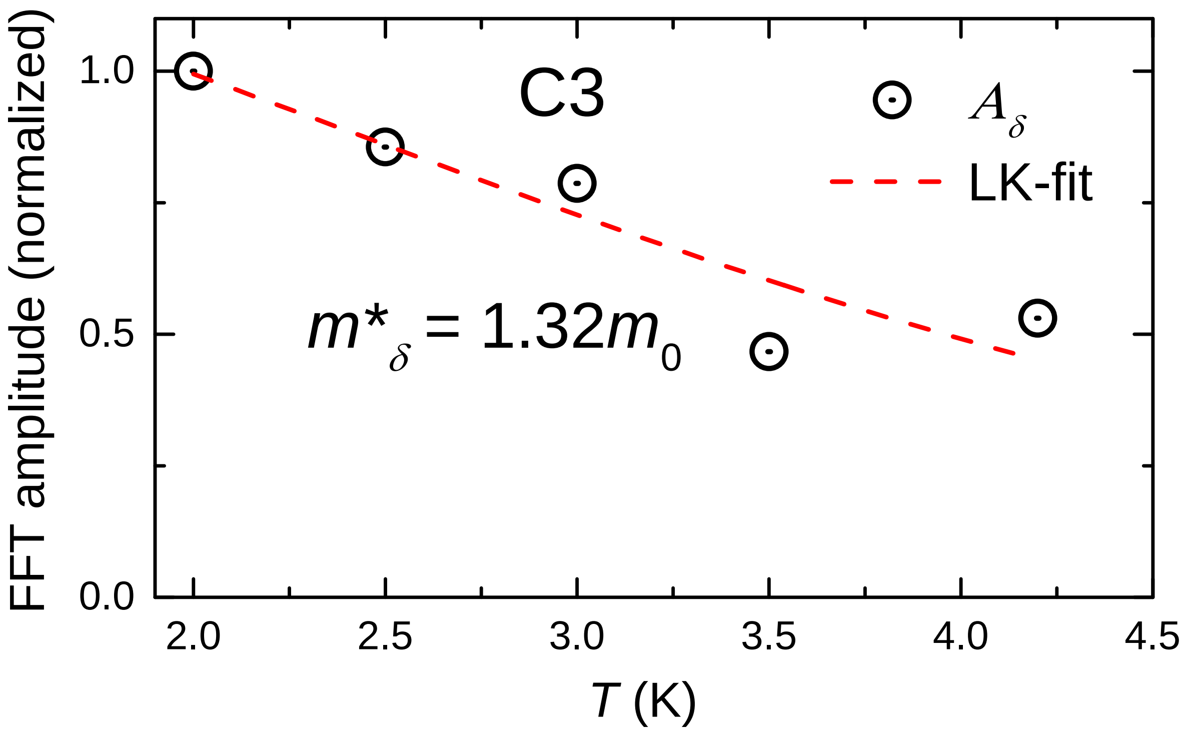

Supplementary Figure 13: Effective mass of the electrons in δ pocket of WP_2. Temperature dependent amplitude variation of the SdH oscillations for the δ electron pocket. The red dashed line is the LK-fit.

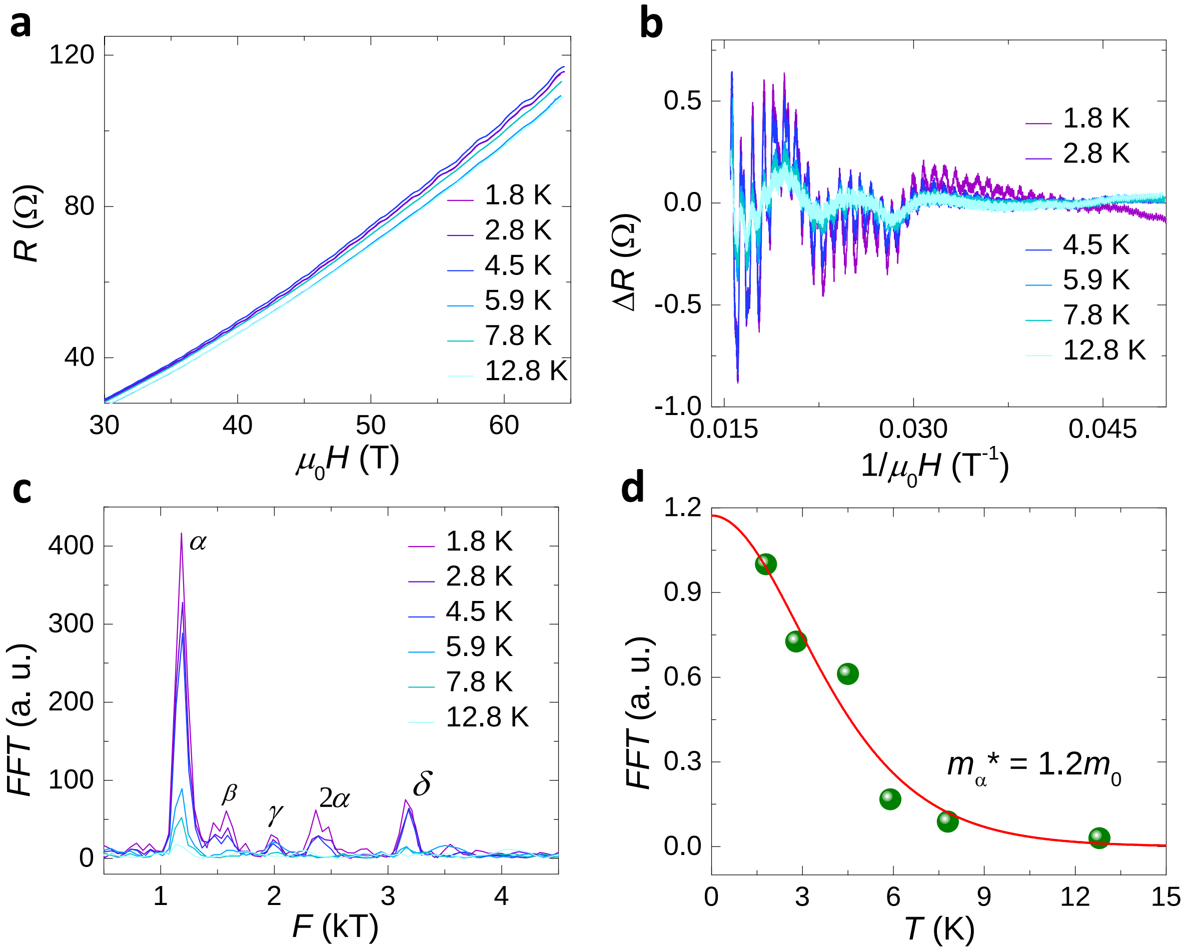

Supplementary Figure 14: SdH oscillations in MoP_2 in magnetic fields up to 65 T. a Resistance at different temperatures from 1.8-12.8 K shows quantum oscillations. **b** corresponding SdH oscillations amplitudes obtained by subtracting a continuous polynomial. **c** FFT amplitudes as a function of the temperature showing the peaks corresponding to holes and electron pockets as predicted by calculations. **d** Effective mass calculations of the α-hole pockets from the LK formula fit to the FFT-amplitude vs *T* data.

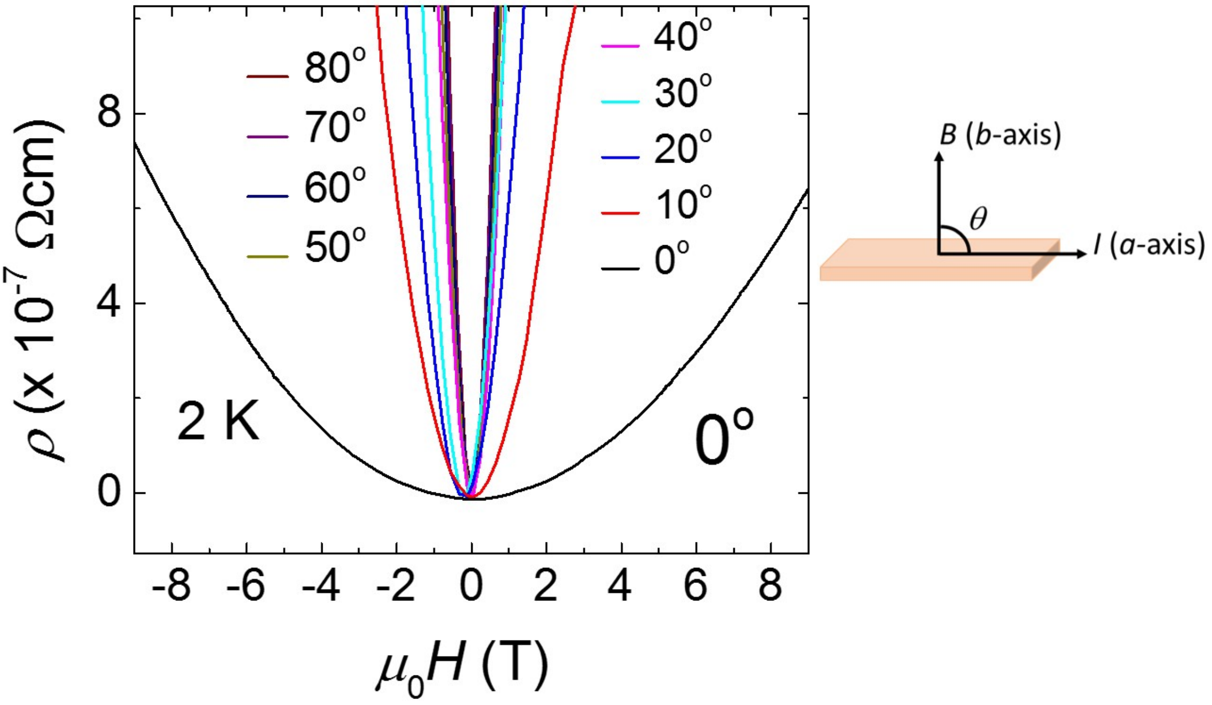

Supplementary Figure 15: Angle dependent *MR* of WP_2 (C4). A large positive *MR* is observed when both electric and magnetic fields are applied along the *a*-axis. The *MR* increases further on increasing the angle between the electric and magnetic field.

References

1. Autès, G., Gresch, D., Troyer, M., Soluyanov, A. A. & Yazyev, O. V. Robust Type-II Weyl Semimetal Phase in Transition Metal Diphosphides XP_2 (X = Mo, W). *Phys. Rev. Lett.* **117,** 066402 (2016)

2. Husmann, A., Betts, J. B., Boebinger, G. S., Migliori, A., Rosenbaum, T. F. & Saboungi, M. L. Megagauss sensors. *Nature* **417,** 421-424 (2002)

3. Ali, M. N., Xiong, J., Flynn, S., Tao, J., Gibson, Q. D., Schoop, L. M.*, et al.* Large, non-saturating magnetoresistance in WTe_2. *Nature* **514,** 205-208 (2014)

4. Kumar, N., Shekhar, C., Wu, S.-C., Leermakers, I., Young, O., Zeitler, U.*, et al.* Observation of pseudo-two-dimensional electron transport in the rock salt-type topological semimetal LaBi. *Phys. Rev. B* **93,** 241106 (2016)

5. Zhao, Y., Liu, H., Yan, J., An, W., Liu, J., Zhang, X.*, et al.* Anisotropic magnetotransport and exotic longitudinal linear magnetoresistance in WT_2 crystals. *Phys. Rev. B* **92,** 041104 (2015)

6. Thoutam, L. R., Wang, Y. L., Xiao, Z. L., Das, S., Luican-Mayer, A., Divan, R.*, et al.* Temperature-Dependent Three-Dimensional Anisotropy of the Magnetoresistance in WTe_2. *Phys. Rev. Lett.* **115,** 046602 (2015)
